# Case Report: Probable Cerebral Amyloid Angiopathy-Related Inflammation During Bevacizumab Treatment for Metastatic Cervical Cancer

**DOI:** 10.3389/fonc.2021.669753

**Published:** 2021-07-27

**Authors:** Tatiana Koudriavtseva, Svetlana Lorenzano, Vincenzo Anelli, Domenico Sergi, Annunziata Stefanile, Enea Gino Di Domenico, Marta Maschio, Edvina Galiè, Carlo Piantadosi

**Affiliations:** ^1^Department of Clinical Experimental Oncology, IRCCS Regina Elena National Cancer Institute, Istituti Fisioterapici Ospitalieri (IFO), Rome, Italy; ^2^Department of Human Neurosciences, Sapienza University of Rome, Rome, Italy; ^3^Department of Research, Advanced Diagnostics and Technological Innovation, IRCCS Regina Elena National Cancer Institute, IFO, Rome, Italy; ^4^Clinical Pathology and Microbiology, IRCCS San Gallicano Dermatologic Institute, IFO, Rome, Italy; ^5^Unità Operativa Complessa (UOC) Neurology, San Giovanni-Addolorata Hospital, Rome, Italy

**Keywords:** case report, bevacizumab, metastatic cervical cancer, cerebral amyloid angiopathy-related inflammation, microhemorrhages 3

## Abstract

Bevacizumab is an anti-angiogenic monoclonal antibody targeting Vascular Endothelial Growth Factor (VEGF) that induces the proliferation and migration of vascular endothelial cells thus, promoting vasculogenesis. Bevacizumab inhibits cancer angiogenesis, which is fundamental for either tumor development, exponential growth, or metastatic spread by supplying nutrients and oxygen. We report a new possible adverse event of bevacizumab, a Cerebral Amyloid Angiopathy-Related Inflammation (CAARI), in a 72-year-old woman with metastatic cervical cancer. After six cycles every three weeks of chemotherapy (cisplatin, paclitaxel, bevacizumab) and following two maintenance bevacizumab administrations, the patient presented a worsening confusional state. The MRI scan showed bilateral asymmetric temporo-parieto-occipital hyperintensity with numerous cortical microbleeds indicative of a CAARI. After stopping bevacizumab treatment, steroid therapy was administered resulting in rapid clinical improvement. The subsequent neurological and oncological follow-up was negative for recurrence. The patient was a heterozygote carrier for apolipoprotein-E *ε*4 that increases the risk of sporadic Cerebral Amyloid Angiopathy (CAA), which is characterized by beta-amyloid accumulation and fibrinoid necrosis in cerebral vasculature leading to micro/macrohemorrhages and dementia. Moreover, CAA is present in 30% of people aged over 60 years without dementia. In the brains of CAA patients, there is a proinflammatory state with cerebrovascular endothelial cell alteration and elevated levels of either adhesion molecules or inflammatory interleukins that increase the blood–brain barrier permeability. Moreover, CAARI is an inflammatory form of CAA. Inhibition of VEGF, which has anti-apoptotic, anti-inflammatory, and pro-survival effects on endothelial cells, impairs their regenerative capacity and increases expression of proinflammatory genes leading to weakened supporting layers of blood vessels and, hence, to damaged vascular integrity. In our patient, bevacizumab administration may have further increased permeability of cerebral microvasculature likely impaired by an underlying, asymptomatic CAA. To our knowledge, this is the first case reporting on the development of probable CAARI during bevacizumab treatment, which should alert the clinicians in case of neurological symptom onset in older patients under anti-angiogenic therapy.

## Introduction

Bevacizumab is a recombinant humanized monoclonal antibody selectively binding to and neutralizing the biologic activity of human Vascular Endothelial Growth Factor (VEGF).

VEGF is a key factor of the angiogenesis physiologically occurring during embryonic development and in adult wound healing. In particular, VEGF induces the proliferation and migration of vascular endothelial cells and therefore, promotes vasculogenesis and angiogenesis by binding two VEGF receptors (VEGFR-1 and VEGFR-2) expressed on vascular endothelial cells. Furthermore, angiogenesis supplies nutrients and oxygen which are fundamental for either cancer development, exponential growth, or metastatic spread (1, 2). Indeed, VEGF is produced by the cancer cells in the “angiogenic switch” and upregulated by either oncogene expression, growth factors, or hypoxia.

Bevacizumab has been available in Europe since 2005 providing multiple indications including cervical advanced cancer (3). It was assumed that bevacizumab inhibits cancer growth supposedly impacting only tumor vessels without damaging other vessels (4). However, in the safety profile of bevacizumab, cerebrovascular events and bleeding are reported among the common adverse events (≥1 and <10%) and hypertensive encephalopathy and reversible posterior leukoencephalopathy syndrome (RPLS) are reported as uncommon adverse events (<1%) (5). There are no cases reported in literature regarding the association between bevacizumab therapy and other encephalopathies such as Cerebral Amyloid Angiopathy (CAA) or CAA-Related Inflammation (CAARI). CAARI is the inflammatory form of CAA that is characterized by beta-amyloid accumulation and fibrinoid necrosis in cerebral vasculature with micro/macrohemorrhages and dementia (6).

To our knowledge, this is the first case reporting on the development of probable CAARI during bevacizumab maintenance monotherapy in a patient affected by metastatic cervical neoplasia. This could be considered as a new side event of bevacizumab, which should alert the clinicians in case of neurological symptomatology onset in older patients under anti-angiogenic therapy.

## Case Description

In November 2016, a 70-year-old Russian female patient with metrorrhagia was diagnosed with a moderately differentiated cervical carcinoma in a Russian hospital. Her previous family and medical history was negative. In that period, no metastases were found. In December, she underwent chemoembolization with cisplatin of the cervical neoformation. In January 2017, she underwent both hysterectoannexectomy and pelvic lymphadenectomy for local recurrence. The scheduled radiochemotherapy was not performed due to the development of a vesicovaginal fistula as a post-surgical complication. After her arrival in Italy in July 2017, the PET/CT scans detected liver and para-aortic and iliac lymph node metastases as well as dubious lung lesions that a subsequent CT total body better defined as pulmonary consolidations of a probable inflammatory nature.

In September, the patient started chemotherapy with bevacizumab, cisplatin, and paclitaxel (six cycles every 3 weeks) with both good tolerance profile and clinical outcome. In fact, in December her CT scan showed a prominent reduction of liver, para-aortic, and iliac lymph node metastases. After the 6th cycle of chemotherapy, the patient had to continue with bevacizumab maintenance therapy but after two administrations of the drug (February 21 and March 14, 2018) she began to experience mild weakness in the right arm and show signs of cognitive impairment.

## Diagnostic Assessment, Therapeutic Intervention, and Follow-Up

**Figure 1:**
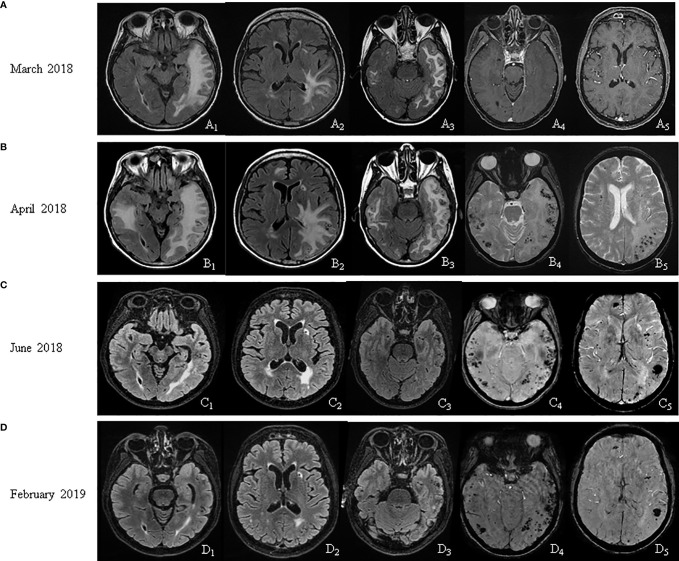
MRI follow up. **(A)** March 2018: FLAIR sequences showed a left temporo-parieto-occipital and a right temporal hyperintensity (A1, A2, A3); no gadolinium enhancement (A4, A5). **(B)** April 2018: FLAIR sequences showed an extension of the white matter (WM) hyperintensity (B1, B2, B3); gradient-echo sequences showed microbleeds (B4, B5). **(C)** June 2018: FLAIR sequences showed a reduction of the WM alterations (C1, C2, C3); susceptibility-weighted imaging (SWI) sequences confirmed microbleeds (C4, C5). **(D)** February 2019: FLAIR sequences showed a further reduction of the WM alterations (D1, D2, D3); SWI sequences corroborated microbleeds (D4, D5).

The brain MRI scan showed a large left temporo-parieto-occipital subcortical and deep white matter hyperintensity (WMH) as well as a small area of WMH in the right inferioromedial temporal lobe on FLAIR sequences (**Figure 1A1-A3**) with no gadolinium enhancement (**Figure 1A4, A5**). Both gradient-echo (GRE) and susceptibility-weighted imaging (SWI) sequences were not performed initially as they are not used as routine procedures in our Institute.

The MRI features resembled a RPLS or hypertensive encephalopathy that may occur as a rare side effect of bevacizumab. However, the blood pressure values of the patient were normal. Routine blood tests together with either inflammation indices such as erythrocyte sedimentation rate, serum autoantibodies (antinuclear antibodies, anti-ssa, -ssb, -sm, -RNP, -scl 70, -Jo1, -ANCA, -MPO, -PR3; IgG, and IgM of either anti-cardiolipin, anti-beta2-glycoprotein I, anti-annexin V, or anti-prothrombin) or serum onconeural antibodies (anti-HU, anti-YO, anti-RI, anti-amphiphysin, anti-CV2, anti-Ma2/Ta, anti-recoverin, anti-sox1, anti-titin, anti-XIC4, anti-GAD65, anti-Tr) tested negative. Virology screening (anti-EBV, anti-herpes simplex 1 e 2, anti-zoster, anti-cytomegalovirus, anti-hepatitis B and C, anti-measles, anti-toxoplasma) was not indicative of ongoing infection. PET/CT scans showed a reduction of focal hypermetabolism in the para-aortic and iliac lymph nodes confirming the findings of the CT total body scan performed in December 2018, along with the disappearance of focal hypermetabolism in the liver. Due to a misunderstanding, the patient did not show up the day she had to perform the EEG.

Over a month, the cognitive impairment of the patient evolved into spatial-temporal disorientation, and then it deteriorated into a confusional state. In April 2018, the MRI revealed an extension of WMH with contralateral involvement, a 4-mm midline shift, and a diffuse venous leptomeningeal stasis (**Figure 1B1-B3**). Numerous cortical microbleeds were also observed on GRE sequences (**Figure 1B4, B5**) mostly in bi-temporal regions. All these findings along with the clinical picture of the patients were suggestive of probable CAARI according to established clinical and radiological criteria. The previous MRI performed in March 2018 was revised in light of these findings, and it actually already evidenced an initial formation of bi-temporal microbleeds, less easily visible on FLAIR sequences (**Figure 1A3**) with their subsequent increase in the month of April (**Figure 1B3**).

Lumbar puncture was not performed for the presence of prominent cerebral edema. However, we had reserved to perform it in case the pathology did not respond to steroid therapy. Therefore, under the hypothesis that this probable CAARI could be related to bevacizumab-induced origin, the drug administration scheduled for April was not performed, and the treatment was interrupted definitely. Intravenous methylprednisolone (120 mg/day for 7 days) was started and was followed by oral prednisone (25 mg/day with gradual tapering over a two week period) leading to rapid improvement of symptoms already after the first few administrations of methylprednisolone. The patient resulted to be heterozygote (*ε*3/*ε*4) for apolipoprotein-E (APOE) *ε*4.

In June 2018, a remarkable reduction of WM lesions with edema resolution was observed (**Figure 1C1-C3**) with persisting multiple microbleeds on SWI sequences (**Figure 1C4, C5**). Consistently with the first MRI (**Figure 1A4, A5**), gadolinium-enhanced lesions were never observed on MRIs at any time-point. In February 2019, patient was cognitively normal while the MRI showed a further reduction of WMH (**Figure 1D1-D3**) with standing microbleeds (**Figure 1D4, D5**).

**Figure 2:**
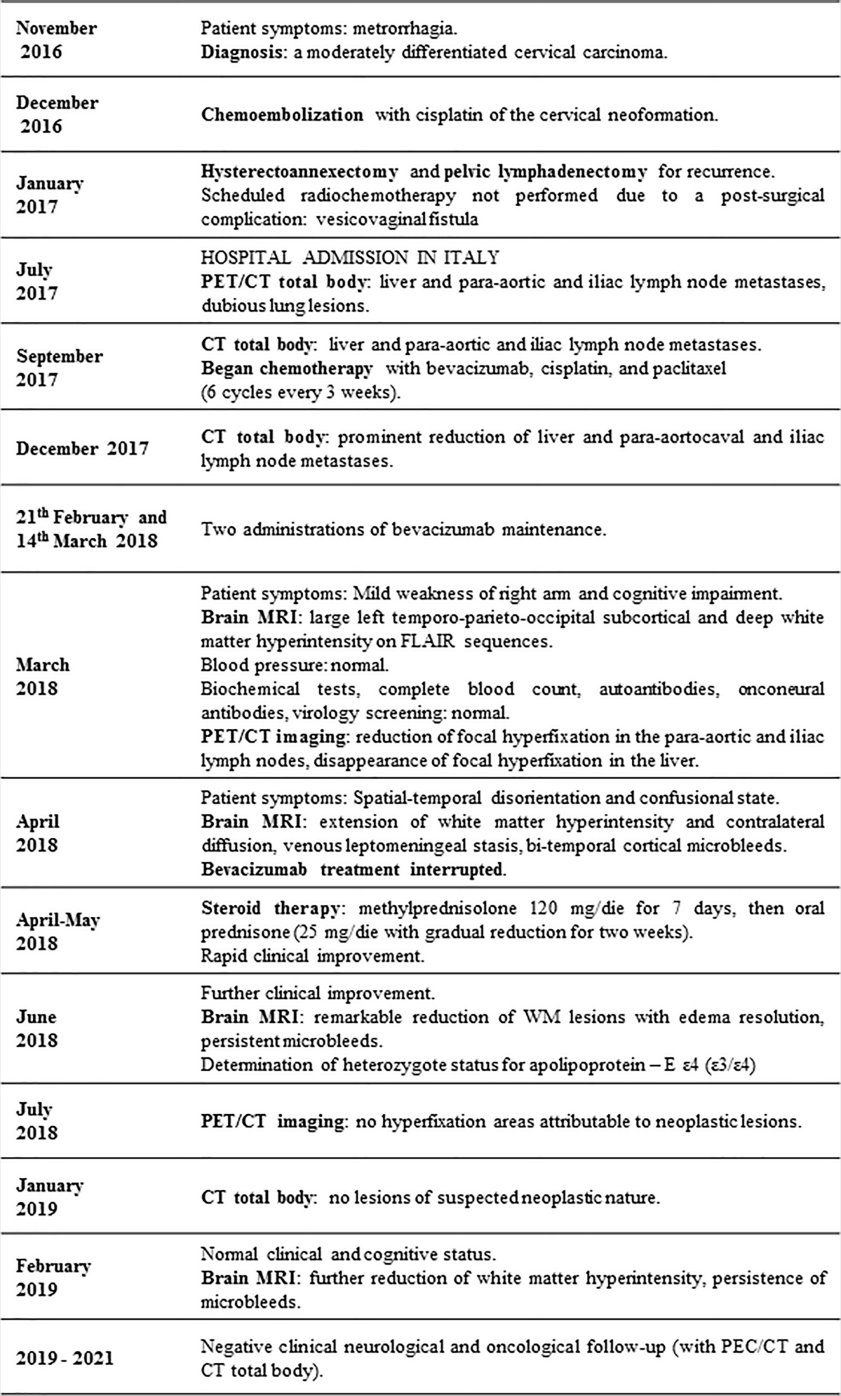
Timeline of clinical course and interventions.

Despite interrupting anti-cancer therapy as early as in April 2018, the PET/CT scans of the patient in July 2018 did not demonstrate areas of hypermetabolism attributable to neoplastic lesions in addition to the CT total body scan performed in January 2019 which did not show any suspicious lesions of neoplastic nature. The subsequent 2-year clinical and radiological oncologic (with CT and PET/CT scans) follow-up was negative for both recurrence and metastasis. The neurological and cognitive status of the patient remained normal. The timeline of the clinical course and interventions of the patient is summarized in **Figure 2**.

## Discussion

This case report is suggestive of a specific inflammatory form of CAA *i.e.*, CAARI, in a patient with a possibly underlying CAA or at least with a predisposition to this disease that the treatment with bevacizumab might have uncovered or acutely worsened. To our knowledge, this is the first study reporting on the potential link between treatment with bevacizumab and the development of CAARI.

The probable diagnosis of CAARI was formed based on the classic or modified clinicoradiological diagnostic criteria (6, 7) since the biopsy was not carried out due to a prompt and complete response to cortisone therapy, whose dosage was even much lower than that widely used (methylprednisolone 1 gr/day for 5 days). Given the optimal response to steroid treatment, it was deemed unethical for our patient to undergo an invasive procedure like brain biopsy even if the pathological examination represents a definitive diagnostic tool for CAARI. In fact, the diagnostic criteria indicate reconsidering a brain biopsy when failure of the empiric high-dose steroid therapy occurs within 3 weeks (6). Moreover, it should be pointed out that in some cases the diagnosis of CAARI could be missed by the biopsy due to sampling error, reflecting the patchy segmental inflammatory nature of this condition. As proof of this, in the study by Aurel et al. aiming to validate the clinicoradiological criteria of probable CAARI, one individual with a negative histological examination was subsequently diagnosed with a probable CAARI according to these criteria, and the diagnosis was confirmed by both clinicoradiological response to steroid therapy and subsequent follow-up (7). Similarly, in one of the largest cohorts of 48 patients with probable CAARI based on clinicoradiological features, only 24 individuals underwent pathologic evaluation, and of these 13% patients had no inflammation (8).

APOE *ε*4 allele, especially in homozygosity (*ε*4/*ε*4), increases the risk not only of sporadic CAA (9) but also of CAARI (6). Our patient has APOE *ε*3/*ε*4 genotype, which is a second more frequent genotype of CAARI (30%) after the APOE *ε*4/*ε*4 one (45%) as reported in some large studies (8).

Other factors that supported our diagnostic hypothesis of CAARI was the age of the patient in the seventh decade, which is the mean age of CAARI onset, as well as her very fast clinical response to steroid therapy (6). However, the most important aspect that guided the diagnostic process was the presence of multiple lobar microhemorrhages (asymmetrically distributed predominantly on the side with more edema) and patchy or confluent asymmetric WMHs on her neuroimaging scans which readily regressed in response to steroid therapy and highly specific for CAARI (8).

CAA is a disorder characterized by beta-amyloid (A*β* peptide) accumulation and fibrinoid necrosis in cerebral vasculature often leading to micro/macrohemorrhages and dementia (10). Furthermore, it is also present in 30% of patients without dementia who are over 60 years old. In the brain of CAA patients, there is a proinflammatory state with cerebrovascular endothelial cell alteration and elevated levels of either adhesion molecules (for example vascular cell adhesion protein 1, intercellular adhesion molecule 1, endothelial leukocyte adhesion molecule-1), inflammatory interleukins or other molecules such as tumor necrosis factor alpha, transforming growth factor beta, monocyte chemoattractant protein-1 that all increase the blood–brain barrier (BBB) permeability (11). A*β* peptide is a derivative from amyloid precursor protein (APP), which is a large type I transmembrane protein (11). APP is expressed in the brain and in peripherally circulating cells such as lymphocytes, monocytes, and platelets. In particular, thrombocytes have very high APP levels contributing to over 90% of the circulating APP. A*β*, a peptide derived and released from APP of activated platelets, produces the formation of vascular amyloid deposits, and this vascular infiltration induces a damage of thinner vessel walls due to a cellular replacement specifically in media and adventitia layers. A*β* peptide also activates and promotes thrombocyte adhesion and aggregation. However, our patient had a normal number of thrombocytes.

CAARI has been reported in a minority of patients with CAA as a newly recognized syndrome of reversible encephalopathy, which presents large areas of inflammation and edema *via* imaging scans (6). Its clinical features were described for the first time by Eng et al. in 2004. The most frequent are: acute/subacute encephalopathy (76%), headache (41%), seizures (31%), and stroke-like signs such as focal neurologic signs (46%) (12). The diagnostic criteria of probable CAARI were defined by Chung et al. in 2011 as follows: acute/subacute onset of symptoms; age ≥40 years; at least one symptom among headache, acute/subacute cognitive decline, mental/behavioral change, focal neurological signs, seizures; MRI showing patchy or confluent asymmetric T2WI or FLAIR WMH (with/without mass effect and with/without leptomeningeal or parenchymal enhancement); recent or past lobar intracerebral hemorrhage and/or multiple cortical/subcortical microbleeds on GRE or SWI; absence of neoplastic, infectious, or other causes (6). Definitive diagnosis of CAARI requires a brain biopsy. The modified clinicoradiological criteria, developed with the aim to spare brain biopsy at least in some cases and compared to the classic diagnostic criteria, have included other possible features useful for diagnosis of probable CAARI with good sensitivity and excellent specificity: 1) clinical symptoms could be also chronic; 2) WMH pattern could extend to the immediate subcortical WM in addition to being asymmetric; and 3) superficial siderosis could be considered as a bleeding manifestation of CAARI (7).

The differential diagnoses include infections (in particular progressive multifocal leucoencephalopathy), neurosarcoidosis, disimmune pathologies and neoplastic processes (primary CNS lymphoma, carcinomatous meningitis, and gliomatosis cerebri) representing, therefore, the conditions to be excluded for the diagnosis of CAARI (6).

Indeed, for our patient, differential diagnosis included RPLS, acute disseminated encephalomyelitis, primary vasculitis of the central nervous system, autoimmune encephalitis, malignancy, and infection. Hypertensive vasculopathy was excluded based on clinical history, examination, and imaging studies that did not demonstrate focal lesions in the basal ganglia, thalamus, cerebellum, and/or brainstem. Even other conditions were excluded based on the negativity of blood chemistry and radiological investigations, especially the prompt response to steroid therapy after discontinuing bevacizumab. Cerebrospinal fluid (CSF) abnormalities such as mildly elevated protein and presence of leukocytes are usually found in CAARI but appear to be neither sensitive nor specific for this diagnosis whereas CSF anti-*β*-amyloid antibodies are not yet commercially available and where their cut-off values are not defined in being variable according to the clinical course (7, 13).

An autoimmune mechanism of inflammation underlying CAARI has been hypothesized for the similarity to the autoimmune inflammation observed after anti-amyloid peptide vaccine and the finding of elevated autoantibodies against A*β* peptide in CSF (14). A case of pathologically confirmed CAARI attributable to anti-PD-1 (Programmed Death-1 receptor) immunotherapy with nivolumab for metastatic melanoma was recently reported as its immune-related adverse event, with a complete response to methylprednisolone therapy (15). PD-1s are important regulators of the threshold of immune response and peripheral immune tolerance that maintain immune homeostasis and prevent autoimmunity. Their inhibition could predispose a proinflammatory state in the brain.

On the other hand, a case of CAARI corroborated through biopsy was described also in a patient with chronic immunosuppression with disseminated mycobacterial infection after orthotopic heart transplantation for sarcoid cardiomyopathy (16). In this case, high-dose intravenous therapy followed by oral steroids led to significant clinical and radiographic improvement.

Therefore, it is important to recognize CAARI early because steroid and immunosuppressive treatment usually leads to clinical and radiological improvement within a few weeks; although CAARI clinical course is variable, improving prognosis and reducing the risk of recurrence is possible (13).

Inhibition of VEGF, which has anti-apoptotic, anti-inflammatory and pro-survival effects on endothelial cells, impairs their regenerative capacity and increases expression of proinflammatory genes leading to weakened supporting layers of blood vessels and hence, to damaged vascular integrity (4). Increased risk of either stroke, transient ischemic attack, or subarachnoid hemorrhage was reported with bevacizumab therapy (4, 5). Predisposition to thrombosis and bleeding under bevacizumab underlines multiple functions of VEGF not only in both endothelial and vascular smooth muscle cells but also in coagulation components (4). Moreover, VEGF increases the production of nitric oxide and prostacyclin having a vasodilator effect; therefore, its inhibition leads to vasoconstriction with both increased peripheral resistance and blood pressure (4). In fact, another rare adverse event of bevacizumab on the brain, an RPLS, which manifests with either BBB breakdown, localized cerebral edema or vasospasm, is ascribed to hypertensive encephalopathy and endothelial dysfunction.

In our case bevacizumab may have been a precipitating factor for CAARI acting on a cerebral microvasculature likely impaired by an underlying, asymptomatic CAA (as suggested by the APOE ε3/ε4 genotype of the patient) through different mechanisms but mainly due to depriving the brain of anti-inflammatory function of VEGF in a CAA-related proinflammatory environment. Moreover, it should be taken into account that beta-amyloid accumulation and fibrinoid necrosis in cerebral vasculature is present in 30% of patients without dementia who are over 60 years old.

## Patient Perspective

This new probable bevacizumab-related adverse event should alert the clinicians in case neurological symptoms develop in older patients under anti-angiogenic therapy and should lead to further investigations *via* performing specific neuroimaging sequences for detecting microbleeds in order to rule out a possible CAARI. In turn, an early diagnosis of CAARI allows for rapidly starting an effective intervention with steroids or, more rarely, immunosuppressive therapy which usually determines a rapid resolution of symptoms. In regard to our patient, prompt steroid therapy resolved acute symptoms, and no neurological worsening was observed during the three years of follow-up. Four and a half years from her diagnosis, our patient has still not experienced any recurrence nor metastasis. Thus, she could fall into the 16.5% of the 5-year survival rate for metastatic cervical cancer (17). She will continue oncological and neurological follow-ups.

## Data Availability Statement

The data analyzed in this study is subject to the following licenses/restrictions: The datasets are available from the corresponding author on reasonable request. Requests to access these datasets should be directed to tatiana.koudriavtseva@ifo.gov.it.

## Ethics Statement

Written informed consent was obtained from the individual(s) for the publication of any potentially identifiable images or data included in this article. The patient has provided her written informed consent for the publication of this case report.

## Author Contributions

TK and CP contributed to the study conception and design. Material preparation, data collection, and analysis were performed by VA, DS, AS, ED, MM, and EG. The first draft of the manuscript was written by SL and all authors commented on previous versions of the manuscript. All authors contributed to the article and approved the submitted version.

## Conflict of Interest

The authors declare that the research was conducted in the absence of any commercial or financial relationships that could be construed as a potential conflict of interest.

## Publisher’s Note

All claims expressed in this article are solely those of the authors and do not necessarily represent those of their affiliated organizations, or those of the publisher, the editors and the reviewers. Any product that may be evaluated in this article, or claim that may be made by its manufacturer, is not guaranteed or endorsed by the publisher.
